# Immobilization of Procerain B, a Cysteine Endopeptidase, on Amberlite MB-150 Beads

**DOI:** 10.1371/journal.pone.0066000

**Published:** 2013-06-11

**Authors:** Abhay Narayan Singh, Sushant Singh, Vikash Kumar Dubey

**Affiliations:** 1 Department of Biotechnology, Indian Institute of Technology Guwahati, Assam, India; 2 Centre for Environment, Indian Institute of Technology Guwahati, Assam, India; Oak Ridge National Laboratory, United States of America

## Abstract

Proteases are involved in several crucial biological processes and reported to have important physiological functions. They also have multifarious applications in different industries. The immobilized form of the enzyme further improves its industrial applicability. Here, we report covalent immobilization of a novel cysteine endopeptidase (procerain B) on amberlite MB-150 beads through glutaraldehyde by Schiff base linkage. The immobilized product was examined extensively by Fourier Transform Infrared Spectroscopy (FTIR), Scanning electron microscopy (SEM) and Energy Dispersive X-ray (EDX) analysis. The characterization of the immobilized product showed broader pH and thermal optima compared to the soluble form of the enzyme. The immobilized form of procerain B also showed lower *Km* (180.27±6 µM) compared to the soluble enzyme using azocasein as substrate. Further, immobilized procerain B retains 38.6% activity till the 10^th^ use, which strongly represents its industrial candidature.

## Introduction

Enzyme immobilization is a process of physical localization of enzymes to a defined surface which helps to improve several enzymatic properties and enhance their operational performance without disturbing their catalytic activity [1]–[6]. Immobilization of enzyme also allow recovery and reusability of the enzyme making the overall process controllable and economical [7]–[8].

Proteases have several applications in food, dairy and detergent industries. It also has a wide range of applicability in medicines, energy production and environmental control [9]–[11]. Industries usually prefer immobilized biocatalysts rather than the traditional chemical methods as it offers reusability, high specificity, easy product separation and negligible byproducts. Due to increasing industrial demand of biocatalysts, different measures are explored to enhance their utilization and reduce their cost. Immobilization proved out to be the best remedy. Proteases are one of the most common industrial enzymes which have wide range of applications varying from food to pharma industries [12]–[15]. Particularly in food industry, the proteases are used in processing of foods. The most common example is the papain from unripe fruits of *Carica papaya* used for meat tenderization [16]. Some neutral and alkaline proteases are used for the recovery of meat from butchering which are used in several canned soups [17]–[18]. Proteases are also used for predigesting glutin protein of wheat in baking as well as dairy industry for production of several dairy products [19]. Certain proteases are used as ingredients of chocolates, cakes and some canned drinks to enhance the flavor.

Proteases from different plants, animals and microbial sources are known. Plant cysteine proteases are well known for their high thermal stability which makes them a potential candidate for several industries where high temperature is required at certain stages [20]. Since the applicability of any protease depends on its functional and stability range and cutting sites, the search for new proteases with unique cutting sites and broad operational range is still continue. We have reported purification and characterized a novel cysteine endopeptidase from the latex of a medicinal plant *Calotropis procera* [21]. The plant is cosmopolitan in nature and belongs to family Asclepiadaceae. It mostly grows as a weed in tropical and sub-tropical regions of Asia and Africa [22]. The plant is well known for its medicinal value and people are using different parts of the plant for the treatment of several diseases from ancient time [23]. Since another cysteine protease (Procerain) is already known from the same source [24], we have named this protease as “Procerain B”. Procerain B has wide functional pH range and retains more than −70% activity till 70°C where most of other enzymes became inactive [25]. We have tested its applicability in food, detergent and dairy industries and found it as a potential candidate for several industrial applications [21]. We have deduced recently the cDNA sequence of this novel endopeptidase which offers the possibility of protein engineering to alter the cleavage specificity and stability of the enzyme. The three dimensional structure of the endopeptidase was also modeled to have some structural information [26]. In order to increase its industrial applicability, we have immobilized procerain B on glutaraldehyde activated chitosan matrix [27] but due to the fragile nature of chitosan beads and leaching problems, we get inspired to have a comparatively stable alternate. In present study, we aimed to immobilize the procerain B on stable amberlite MB-150 beads with glutaraldehyde and characterize the immobilization product.

## Materials and Methods

### Materials

The enzyme was purified from the latex of *Calotropis procera* by the method of *Singh et al., 2010* [25]. CM-Sepharose FF was purchased from GE Healthcare. The amberlite MB-150 beads, azocasine, glutaraldehyde, protease inhibitor, trichloroacetic acid (TCA), Sodium tetrathionate (STT) and Bradford reagent were purchased from Sigma Chemicals Co. (St. Louis, MO). Sodium chloride, Tris-HCl buffer, dialysis tubing, β-mercaptoethanol (β-ME) were purchased from Merck Milipore (Germany). All other chemical were of highest purity commercially available. All reagents were prepared in Milli Q water (Millipore, United State).

### Methods

#### Determination of protein concentration

The protein concentration was determined at different steps of immobilization by the method of Bradford with BSA as standard [28].

#### Determination of protease activity

The proteolytic activity of procerain B was determined as described earlier [21], [24], with azocasein and hemoglobin as substrate. The enzyme (5 µg) was incubated at 37°C for 10 min in 500 µl of Tris-HCl buffer pH 7.5 containing 50 mM β-Mercaptoethanol as reducing agent. Azocasein solution (1%) was prepared in same buffer without β-Mercaptoethanol and added in enzyme solution to make the final volume 1 ml. The solutions were mixed properly and incubated at 37°C for 30 min. TCA (10%) was added to the reaction mixture to stop the reaction and incubated at room temperature for 5 min. The mixture was centrifuged at 10,000 rpm for 10 min. In case of azocasein as substrate, 500 µl of supernatant was mixed with equal volume of 50 mM NaOH and the color developed was quantified spectrophotometrically at 440 nm. A control assay was done without enzyme and used as blank in all spectrophotometric experiments. In case of hemoglobin as substrate, the supernatant after TCA precipitation was quantified directly at 280 nm. For the determination of protease activity of immobilized enzyme, 50 mg of amberlite beads with immobilized procerain B were used in all experiments.

#### Immobilization of enzyme

Procerain B was immobilized on glutaraldehyde activated amberlite MB-150 beads (100–200 mg) of diameter 0.5 mm. Before activation with glutaraldehyde, the beads were equilibrated for overnight at different pH in the range of pH 4 to 10. The buffers used for equilibration at different pH were, acetate buffer (50 mM) for pH 4, phosphate buffer (50 mM) for pH 6, Tris-HCl buffer (50 mM) for pH 8 and pH 10-. The equilibrated beads were treated with different glutaraldehyde concentrations in the range of 1% to 5%v/v at room temperature for different time intervals and then washed extensively with respective buffers to remove the excess of glutaraldehyde. The complete removal of unreacted glutaraldehyde was confirmed spectrophotometrically by monitoring the absorbance of wash, till the absorbance was lower than 0.01 at 280 nm. In order to determine the appropriate time for coupling reaction, the activated beads were incubated with enzyme for different time intervals and the coupling time which corresponds to maximum immobilization was used for further experiments. The activated beads were incubated with different concentrations of purified enzyme and the optimum concentration at which maximum immobilization was achieved was considered for further experiments.

The immobilization (percentage enzyme activity on beads) was calculated as follows:
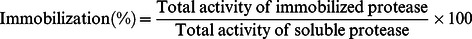



**Note:** Total activity of immobilized protease was determined by subtracting total activity of unbound protease from total activity of soluble enzyme.

#### Fourier Transform Infrared Spectra (FTIR) of amberlite MB-150 beads

The activation of amberlite beads with glutaraldehyde was confirmed by comparing the FTIR spectra of normal and glutaraldehyde activated beads. The FTIR spectra were taken with UNICAM Mattson1000 FTIR Spectrophotometer and compared with each other. In order to prepare the sample for FTIR analysis the beads were crushed with potassium bromide (KBr) and compressed in form of thin pellet which was used directly for the analysis.

#### Scanning Electrom Microscopy (SEM) and Energy Dispersive X-ray (EDX) analysis of amberlite beads

The detailed surface morphology and micro structural details of normal, activated and immobilized amberlite MB-150 beads were analyzed by SEM. The samples were coated with gold to make the surface conducting and then imaged and photographed by secondary electron imaging by SEM (LEO 1430 VP) at an acceleration voltage of 10.00 kV. The EDX spectra were also collected to analyze the elemental composition of bead surface.

#### pH and temperature optima of immobilized procerain B

The activity of immobilized procerain B was studied as a function of pH to determine the optimum pH of immobilized enzyme. In all activity assay experiments the amount of amberlite beads were kept constant (50 mg). The buffers used for different pH were, 50 mM glycine-HCl (pH 2–3.5), 50 mM acetate buffer (4–5.5), 50 mM phosphate buffer (pH 6–7.5), 50 mM Tris-HCl buffer (pH 8–10), 50 mM Na-carbonate buffer (pH 10.5–12). The substrate solutions (1% azocasein or hemoglobin (w/v) were prepared in respective buffers. The beads with immobilized procerain B were incubated in respective buffers for 15 min and then the activity assay was performed as described earlier. Hemoglobin was used as substrate at pH less than 4.0 due to insolubility of azocasein at lower pH [29].

The effect of temperature on the activity of immobilized procerain B on amberlite beads were studied in the range of 10–95°C. Immobilized amberlite beads (50 mg) were incubated at different temperatures for 15 min in Tris-HCl buffer pH 8 and then assayed for proteolytic activity at corresponding temperatures as described earlier. The 1% azocasein solutions in same buffer were already incubated at respective temperatures and used as substrate during experiment. A control assay without enzyme was performed at each temperature and used as blank.

#### Stability of immobilized procerain B

The effect of pH (2–12) and temperature (10–95°C) on the stability of amberlite immobilized procerain B were studied in terms of residual activity. Immobilized amberlite beads (50 mg) were incubated at different pH for overnight at room temperature and the residual activity was tested by activity assay as described earlier with azocasein as substrate.

Similarly the effect of temperature was also studied by incubating 50 mg of immobilized amberlite beads at different temperatures for 15 min and then testing the residual activity with azocasein as substrate at 37°C.

### Kinetic Parameters

Kinetic parameters for immobilized procerain B were tested at pH 8 and 37°C with azocasein as substrate by increasing the concentration from 10 to 400 µM. For each concentration, a control reaction was done without enzyme. The Michaelis-Menten constant (K_m_) and reaction velocity (V_max_) were calculated by Lineweaver-Burk plot.

### Reusability of Immobilized Procerain B

The reusability of immobilized procerain B on amberlite beads were tested by repeated use of same amberlite beads. The activity assay was done as described earlier and after every use, the beads were washed with Tris-HCl buffer pH 8 and reused for next batch of reaction.

## Results and Discussion

Enzymes are the biocatalysts which enhance the rate of a reaction. In order to meet the huge demand of exponentially increasing population, industries are exploring several enzymes for different purposes [30]. Use of normal soluble enzymes increases the industrial enzymatic demand which cannot be fulfilled with limiting resources. The only alternative is the use of immobilized enzymes, which can limit the enzymatic demand by its repeated use. A variety of matrices are available for immobilization of enzymes and each has its own advantages and disadvantages [31]. Amberlite is a stable and comparatively robust matrix which can be used for immobilization purpose. We have purified a novel cysteine protease and proved its importance in several industries [21], [25]. In present study we are focusing on immobilization of procerain B on stable and robust amberlite MB-150 beads and characterization of immobilized product for industrial use, which will further enhance the industrial importance of this enzyme. Here we have optimized the immobilization of procerain B on amberlite beads by covalent attachment through glutaraldehyde, which was used as a linker.

### Effect of pH on Immobilization of Procerain B

**Figure 1:**
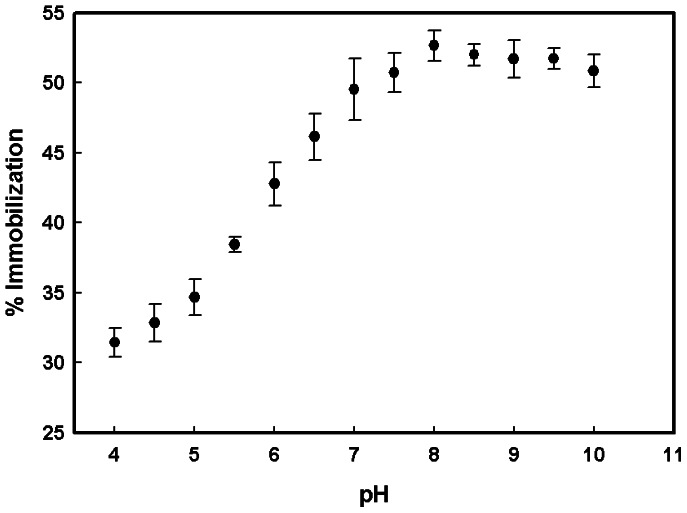
Effect of pH on immobilization of procerain B on glutaraldehyde activated Amberlite MB-150 beads in the range of pH 4–10. The optimum pH for immobilization of procerain B was nearly 8.0 with 52.65% immobilization.

The amberlite beads are the mixture of cationic and anionic resins, so in order to find a suitable pH for immobilization of procerain B, the overnight equilibrated beads at different pH were activated with glutaraldehyde (2%, v/v) for 6 h. The activated beads were washed extensively with respective buffers and then incubated for 24 hr with procerain B for immobilization. The maximum immobilization (52.65%) was observed at pH 8 **(** **Figure 1** **)**. At lower pH the immobilization was comparatively less, which may be due to improper ionization state at the surface of amberlite beads.

### Optimization of Immobilization on Amberlite MB-150 Beads

The immobilization of procerain B on amberlite MB-150 beads was optimized in terms of glutaraldehyde concentration, activation time, coupling time and enzyme (Procerain B) concentration. Different glutaraldehyde concentrations (1–5%) were used to activate the beads for immobilization of enzyme and 4% was found to be optimum with 54.51±0.62% immobilization **(** **Table 1** **)**. The time for activation with 4% glutaraldehyde concentration was also optimized in the range of 1–8 h and 4 h was found to be most effective (55.86±0.58% immobilization) for amberlite activation **(** **Table 1** **)**. However, the activation for more than 4 h did not show any further increase in immobilization. The next parameter to be optimized was coupling time for attachment of enzyme through free amino group with aldehyde of glutaraldehyde activated amberlite beads. The coupling time was varied from 8–32 h and 24 h was the most effective with 56.98±0.72% immobilization **(** **Table 1** **)**. For economic use of enzyme in immobilization the enzyme concentration was also optimized by varying the concentration from 0.2 mg/ml to 1.0 mg/ml. Maximum immobilization (62.07±1.13%) was achieved with 0.8 mg/ml enzyme concentration **(** **Table 1** **)**.

**Table 1:** Optimization of immobilization conditions for procerain B on gluteraldehyde activated Amberlite MB-150 beads. Data provided in the table are the average of three independent experiments and the bold figures show the optimum results obtained.

	GlutaraldehydeConcentration (%)	ActivationTime (hr.)	CouplingTime (hr.)	Protein Concentration inImmobilization mixture(mg/ml)	Immobilization(%)
**Variation of glut. concentration**	1	6	12	0.2	49.56±0.43
	3	6	12	0.2	51.39±1.90
	4	6	12	0.2	52.66±2.71
	5	6	12	0.2	**54.51±0.62**
		6	12	0.2	51.89±0.83
**Variation of activation time**	4	1	12	0.2	46.15±2.38
	4	2	12	0.2	50.31±1.25
	4	4	12	0.2	**55.86±0.58**
	4	6	12	0.2	54.19±0.16
	4	8	12	0.2	53.93±0.87
**Variation of coupling time**	4	4	8	0.2	43.55**±**0.35
	4	4	16	0.2	56.30±0.79
	4	4	24	0.2	**56.98**±**0.72**
	4	4	32	0.2	55.62±1.53
**Variation of protein concentration**	4	4	24	0.2	57.05±0.36
	4	4	24	0.4	58.23±1.35
	4	4	24	0.6	60.18±1.53
	4	4	24	0.8	**62.07±1.13**
	4	4	24	1.0	60.86±1.71

**Figure 2:**
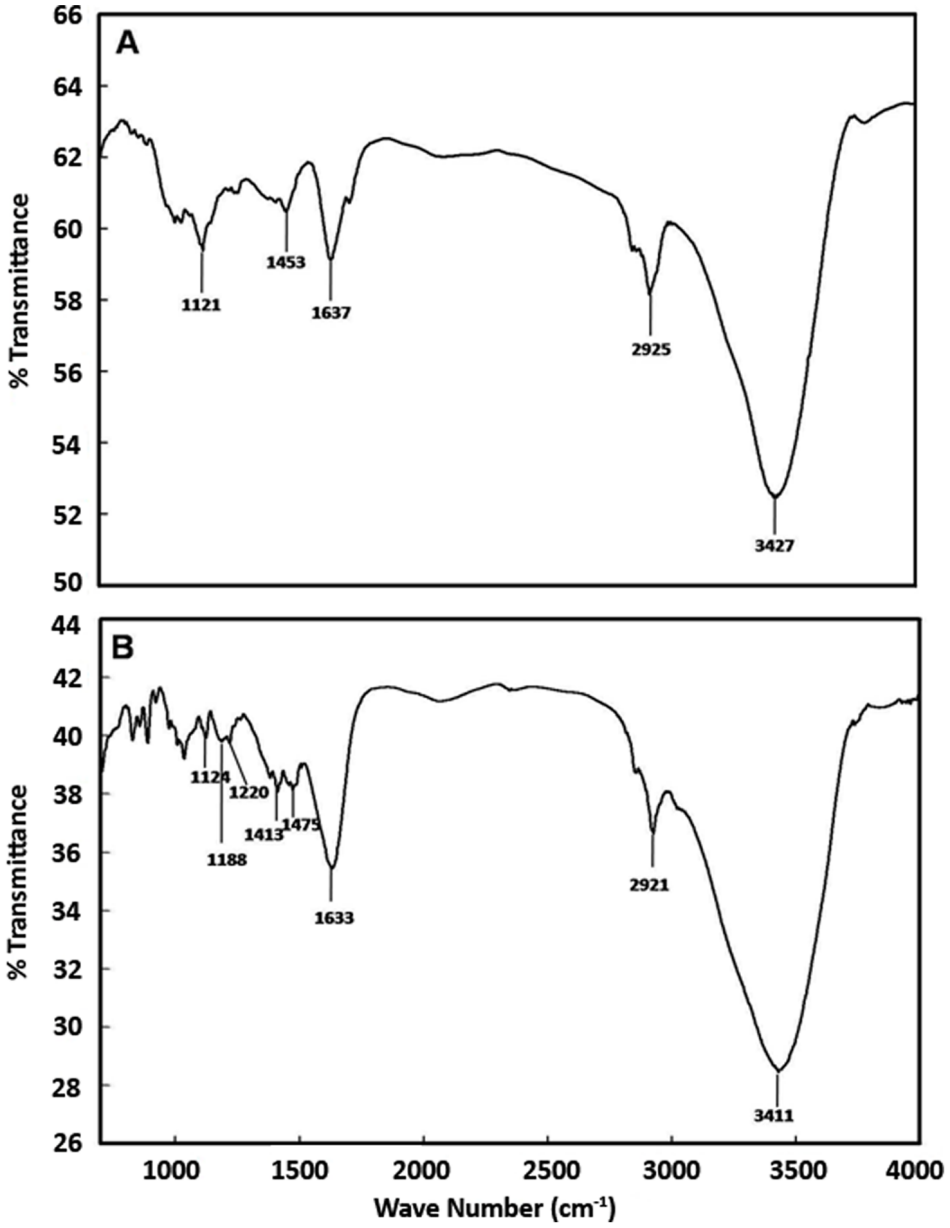
Comparison of FTIR spectra of normal and glutaraldehyde activated Amberlite beads. For FTIR analysis the beads were crushed with KBr and compressed to form a thin pellet. The pellet was used for FTIR analysis. (**A**) FTIR spectra of normal Amberlite beads. (**B**) FTIR spectra of glutaraldehyde activated Amberlite beads. Both spectra were compared for confirmation of glutaraldehyde activation of beads. The peaks at 2925, 1453 and 1121 are due to amberlite. The increase in 1637 peak intensity is due to activation of bead with glutaraldehyde.

**Figure 3:**
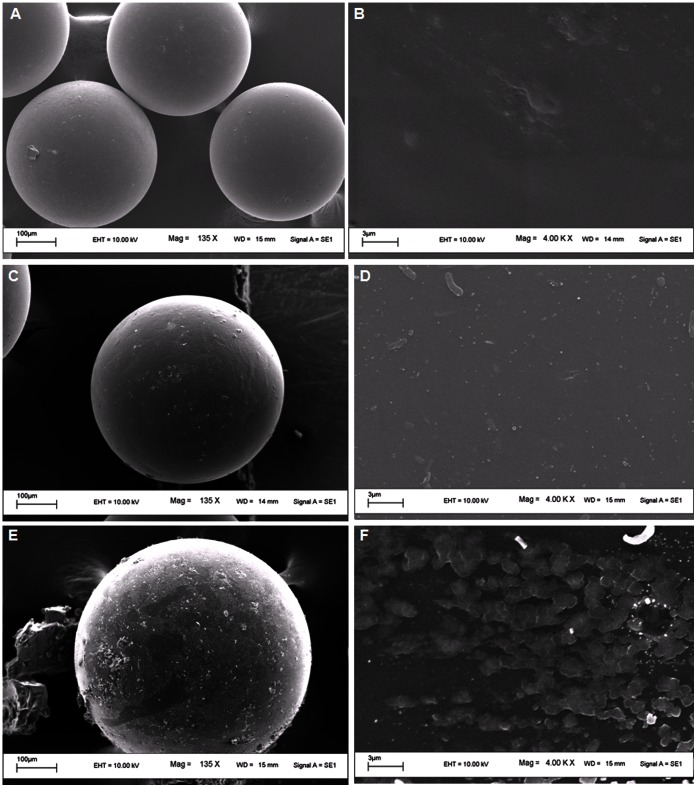
SEM images of beads. The detailed surface view of (**A and B**) normal Amberlite beads, (**C and D**) glutaraldehyde activated Amberlite beads, (**E and F**) immobilized Amberlite beads.

The activation of amberlite MB-150 beads with 4% (v/v) glutaraldehyde concentration was confirmed by Fourier Transform Infrared Spectra (FTIR). The spectra of normal and activated beads were compared. The peaks at 3427 cm^−1^, 2925 cm^−1^ and 1121 cm^−1^ in normal amberlite beads **(** **Figure 2** **, A)** was due to amberlite composition and the corresponding peaks were also present in glutaraldehyde activated beads at 3411 cm^−1^, 2921 cm^−1^ and 1124 cm^−1^ respectively **(** **Figure 2** **, B)**. The peak at 3427 cm^−1^ corresponds to O-H stretch of alcohol, 2925 cm^−1^ represents alkane C-H stretch and 1121 cm^−1^ reflects the C-N stretch of amine. The increased peak intensity at 1633 cm^−1^ in graph B might be due to activation of beads with glutaraldehyde. Other peaks at 1453 cm^−1^ in graph-A, 1413 cm^−1^ and 1475 cm^−1^ in graph-B were due to aromatic C = C stretch whereas the peak at 1220 cm^−1^ in graph B represents C-O stretch of acid. The SEM analysis of beads represents the changed topology of activated and immobilized beads in comparison to normal amberlite beads **(** **Figure 3** **)**. The Energy Dispersive X-ray (EDX) analysis by area scan suggests increased nitrogen percentage on the surface of immobilized beads **(Material S1)** in comparison of normal and activated one, which may be due to immobilization of procerain B on the surface of amberlite beads.

### Characterization of Immobilized Procerain B

**Figure 4:**
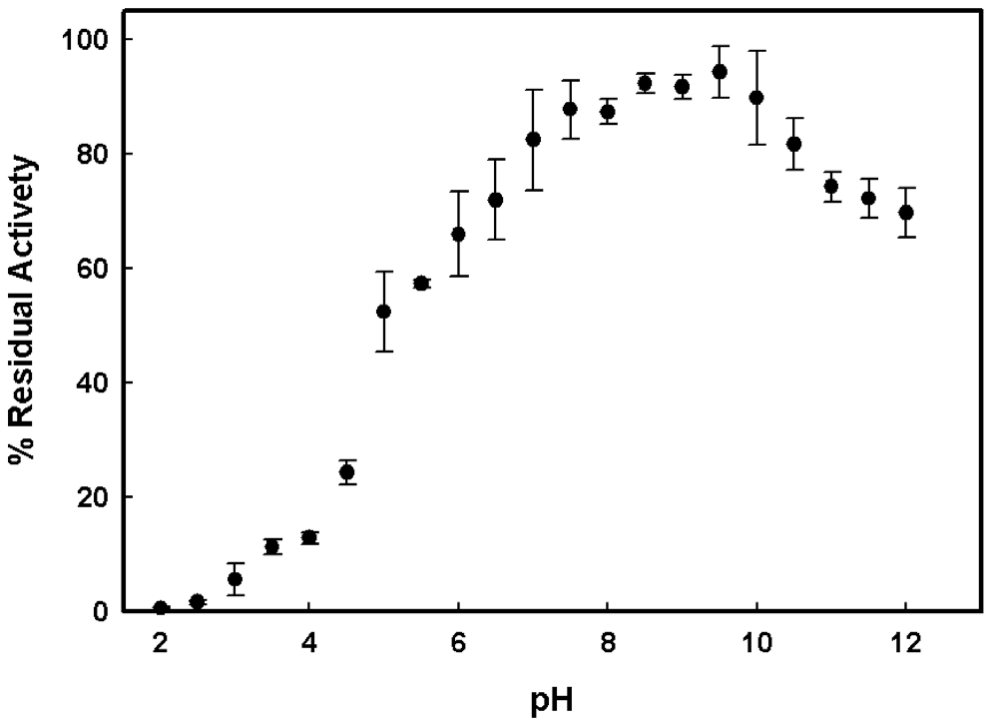
Effects of pH on activity of immobilized procerain B. For the effect of pH on activity the substrate was also prepared in the buffers of respective pH. Stability was determined by overnight incubating the enzyme at room temperature at different pH conditions and next day activity was taken as described in method section.

**Figure 5:**
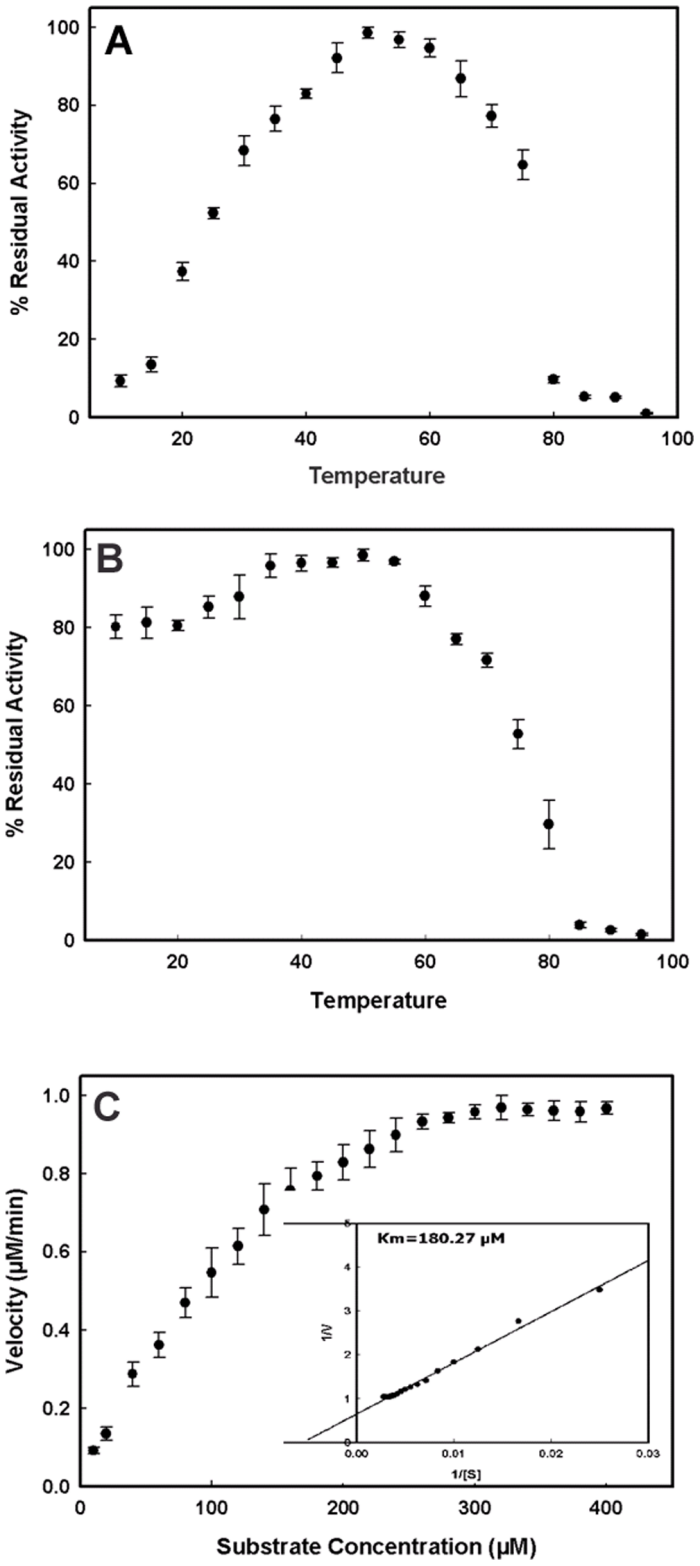
Biochemical characterization of immobilized procerain B. (**A**) Effect of temperature on activity of immobilized procerain B at pH 7.5, the substrate was also pre-incubated at respective temperatures and reactions were also carried at respective temperatures. (**B**) Effect of temperature on the stability of immobilized enzyme. For stability measurements, the enzyme was first incubated at required temperature for 15 min and then, the activity was measured at 37°C and pH 7.5. (**C**) Effect of substrate concentration on reaction velocity of immobilized procerain B. The Km value for azocaseine as substrate was calculated from the Lineweaver-Burk plot showed in subset of the graph.

In comparison to soluble procerain B [25] the pH optima of enzyme was shifted toward alkaline pH after immobilization on amberlite MB-150 beads. The immobilized enzyme showed pH optima in range of 8.5 to pH 10 **(** **Figure 4** **)**. It can be used in the reactions where alkaline pH is required. The functional thermal optima of procerain B immobilized on the surface of amberlite beads was found to be in the range of 45–65°C **(** **Figure 5A** **)** which is higher than the soluble form [25]. Increased thermal optima make it suitable for applications with higher temperature.

Temperature stability of an enzyme is a prime concern especially for enzymes with industrial importance as same enzyme is going to be used for different batches of reactions. In terms of thermal stability, the immobilized procerain B retains its maximum activity (more than 80%) up to 60°C **(** **Figure 5B** **)**. Procerain B immobilized on amberlite beads follows Michaelis-Mentan kinetics with increasing substrate concentration. The Lineweaver-Burk plot showed an apparent Km of 180.27±6 µM for azocasein as substrate **(** **Figure 5C** **)** which is less than soluble form of enzyme (210 µM). Decrease in Km reflects the enhanced affinity of amberlite immobilized enzyme for substrate in comparison to soluble form. This might be due to conformational change in enzyme after immobilization or increased localized concentration of substrate around beads due to particular ionic state of beads at that pH.

### Reusability of Immobilized Procerain B

**Figure 6:**
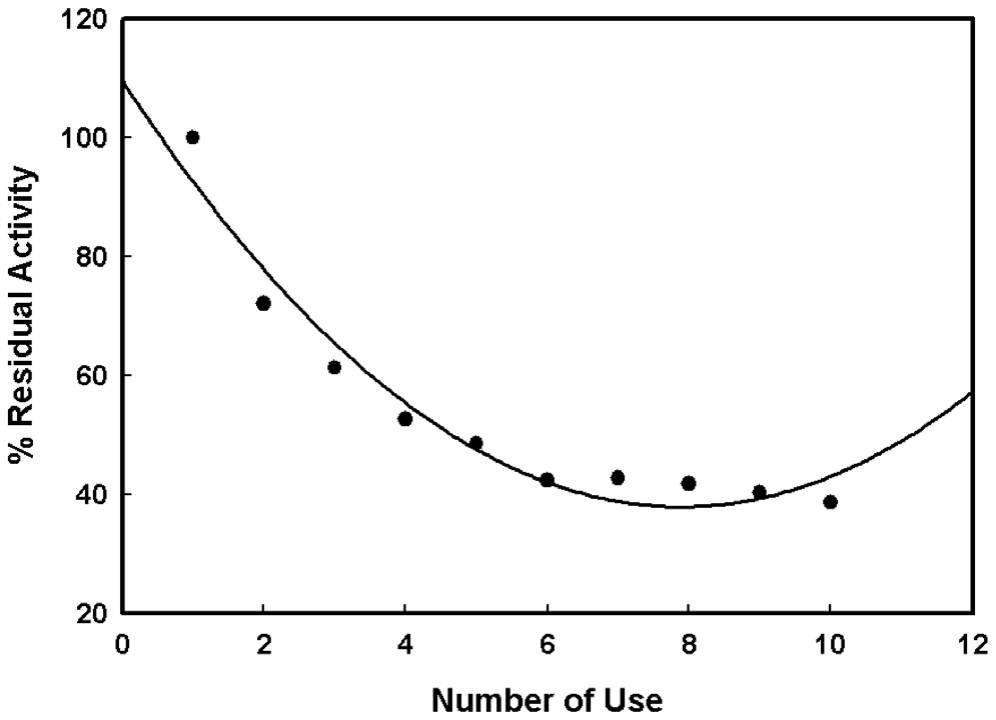
Reusability of procerain B immobilized on amberlite MB-150 beads. The reusability of was tested by repeated use of same amberlite beads. After every use the beads were washed with Tris-HCl buffer pH 8 and reused for next batch of reaction.

Reusability of immobilized enzyme is one of the important advantages which makes the immobilized enzyme a preferred choice over the soluble form. The procerain B immobilized on amberlite beads showed 38.60% activity till 10^th^ batch of reaction **(** **Figure 6** **)**.

### Conclusion

Amberlie MB-150 is a robust ionic resin for immobilization of procerain B. After glutaraldehyde activation nearly 62% immobilization of procerain B can be achieved with optimized parameters. The immobilized form of enzyme showed comparatively broad pH and temperature optima with better stability in alkaline pH range which proves its candidature as a potential candidate for different industrial purpose.

## Supporting Information

Material S1 **EDX analysis of bead surface.** A. Normal Amberlite MB-150 beads, B. Glutaraldehyde activated Amberlite MB-150 beads, C. Immobilized Amberlite MB-150 beads.
(DOC)
