# Supplementary material for: Immobilization of Procerain B, a Cysteine Endopeptidase, on Amberlite MB-150 Beads
Source: PLoS One. 2013 Jun 11;8(6):e66000. doi: 10.1371/journal.pone.0066000 (PMC3679035; doi:10.1371/journal.pone.0066000)
Supplement: Material S1 — EDX analysis of bead surface. A. Normal Amberlite MB-150 beads, B. Glutaraldehyde activated Amberlite MB-150 beads, C. Immobilized Amberlite MB-150 beads. (DOC) [file pone.0066000.s001.doc]

**Material S1**

| **Atomic % of**  **elements** | **A** | **B** | **C** |
| --- | --- | --- | --- |
| **C** | **66.19** | **56.52** | **60.86** |
| **N** | **10.07** | **10.42** | **21.23** |
| **O** | **21.35** | **26.66** | **17.62** |
| **Others** | **2.39** | **6.39** | **0.3** |

**^Note^:** ^C = Carbon, N = Nitrogen, O = Oxygen^.
